# Supplementary material for: The Role of Reproductive Modes in Shaping Genetic Diversity in Polyploids: A Comparative Study of Selfing, Outcrossing, and Apomictic Paspalum Species
Source: Plants (Basel). 2025 Feb 6;14(3):476. doi: 10.3390/plants14030476 (PMC11820972; doi:10.3390/plants14030476)
Supplement: Supplementary file 1 [file plants-14-00476-s001.zip › plants-3394717-supplementary.pdf]

Table S1. Coefficients of Variation of each phenotypic trait (in percentages).

Species	Pop	n	H	Hw	LL	LW	ShL	NL	IL	BRL	EBR	NR	VL	SL	SW	VP	FP
P. intermedium	PIN2	14	9.1	9.1	12.4	4.7	11.2	12.3	5.8	11.0	8.8	6.3	6.7	6.8	6.2	11.3	4.9
	PIN4	18	7.5	7.4	12.0	4.2	14.0	10.0	6.1	10.0	24.9	12.7	5.7	2.0	0.0	10.8	4.5
	PIN7	18	19.2	19.7	12.5	10.9	8.2	**37.4**	10.7	9.7	8.0	15.3	18.3	2.2	0.0	11.1	5.9
	PIN8	18	18.0	11.6	20.9	21.8	18.6	15.3	13.1	18.7	20.3	10.4	17.9	5.1	5.9	13.7	12.8
	PIN9	18	11.4	13.8	26.5	23.9	11.1	18.3	13.6	14.2	13.6	6.6	9.1	2.9	5.3	9.1	5.1
P. durifolium	PD3	16	14.8	11.9	26.3	14.5	24.7	22.8	12.1	11.8	13.2	17.2	14.6	1.7	4.4	9.9	8.3
	PD4	16	19.1	10.6	15.1	14.3	22.2	**32.1**	12.6	15.0	17.4	20.8	21.6	4.2	4.1	14.1	8.9
	PD5	19	14.7	10.7	20.2	14.5	22.2	23.8	12.3	14.4	17.7	15.3	15.0	3.2	4.8	6.9	6.4
	PD1	19	18.3	16.5	19.1	12.5	25.1	**34.4**	11.0	17.8	20.3	22.9	18.9	4.4	8.6	9.4	6.8
	PD2	16	12.0	19.9	19.3	11.5	14.7	20.2	12.7	17.3	15.8	16.5	12.1	3.5	4.5	8.2	5.6
P. ionanthum	PI3	19	19.3	10.9	17.1	11.3	16.3	20.5	16.6	15.0	17.6	9.1	22.3	6.8	1.7	13.7	7.6
	PI4	20	15.5	10.3	16.5	8.2	18.2	18.2	12.0	14.5	20.4	18.1	15.6	8.0	0.0	16.6	9.7
	PI5	19	16.6	14.7	11.5	13.3	23.4	18.5	13.5	14.3	15.5	12.3	18.0	6.3	2.5	11.3	9.6
	PI1	19	6.9	11.4	11.3	10.0	17.6	9.8	11.9	11.0	15.4	3.4	3.8	4.9	1.6	20.9	12.5
	PI2	19	20.4	10.6	17.7	12.3	20.5	29.3	15.1	16.4	14.5	3.4	19.8	5.5	7.00	11.9	9.1
P. regnellii	PR2	18	7.0	8.2	15.3	9.5	21.2	13.7	14.7	11.7	16.1	13.4	9.9	0.0	0.0	12.0	1.5
	PR1	20	6.3	2.9	10.4	10.6	14.3	11.1	15.4	10.7	16.2	20.9	10.3	0.0	0.0	7.6	1.1
	PR3	20	5.9	9.9	15.7	11.6	11.8	10.6	16.6	15.1	20.8	20.7	9.4	0.0	0.0	12.4	3.4
	PR4	20	6.6	7.1	15.2	10.6	15.7	15.8	15.9	13.6	15.3	19.5	8.2	0.0	0.0	8.8	2.6
	PR5	19	4.3	21.6	14.5	6.7	10.8	12.0	15.8	10.7	14.4	24.6	6.9	0.0	0.0	12.2	3.0
P. urvillei	PU1	16	4.2	7.7	11.8	5.8	9.9	10.5	17.3	17.2	17.2	24.8	10.3	3.1	0.0	23.5	5.1
	PU3	18	5.2	8.1	7.5	12.1	11.9	15.3	11.3	16.7	15.9	18.9	7.1	5.0	0.0	**30.2**	18.0
	PU4	17	7.3	8.2	15.7	11.1	11.2	10.3	10.1	10.3	10.3	10.2	8.9	3.7	0.0	**41.0**	21.0
	PU5	17	5.9	9.1	14.2	13.9	11.6	14.2	13.5	18.6	14.7	13.3	10.0	5.0	0.0	**34.7**	27.2
	PU2	20	6.8	10.5	12.2	14.4	10.3	13.6	20.4	18.7	11.7	18.9	9.2	6.9	0.0	**36.2**	21.9

References: n, sample size; *H*, plant height with flowering culms; *Hw*, plant height without flowering culms; *LL*, 2nd leaf blade length; *LW*, 2nd leaf blade width, *ShL*, 2nd leaf sheath length; *NL*, 2nd internode length; *IL*, inflorescence length; *BRL*, basal raceme length; *EBR*, number of spikelets in the basal raceme; *NR*, raceme number; *VL*, flowering culms length with inflorescences; *SL*, spikelet length; *SW*, spikelet width; V*P*, extension of the vegetative period; *FP*, extension of the flowering period. Numbers in red indicate a %CV >30.

Table S2. Kruskal-Wallis Test.

Species	variable	n	statistic	d.f.	p-value
P. intermedium					
	BRL	86	39.4	4	<0.001
	EBR	86	33.5	4	<0.001
	FP	86	75.4	4	<0.001
	H	86	65.3	4	<0.001
	Hw	86	65.4	4	<0.001
	IL	86	62.0	4	<0.001
	LL	86	49.1	4	<0.001
	LW	86	44.6	4	<0.001
	NL	86	66.1	4	<0.001
	NR	86	73.5	4	<0.001
	SL	86	65.9	4	<0.001
	ShL	86	46.0	4	<0.001
	SW	86	79.1	4	<0.001
P. durifolium					
	FP	86	19.0	4	0.001
	LL	86	17.5	4	0.002
P. ionanthum					
	FP	96	39.0	4	<0.001
	H	96	26.5	4	<0.001
	LL	96	23.4	4	<0.001
	NL	96	17.0	4	0.002
	SL	96	20.0	4	<0.001
	ShL	96	59.7	4	<0.001
	SW	96	47.0	4	<0.001
P. regnellii					
	EBR	97	15.7	4	0.003
	FP	97	32.9	4	<0.001
	H	97	20.6	4	<0.001
	Hw	97	37.3	4	<0.001
	ShL	97	22.7	4	<0.001
	VL	97	16.9	4	0.002
	VP	97	33.5	4	<0.001
P. urvillei					
	FP	88	14.1	4	0.007
	H	88	25.1	4	<0.001
	Hw	88	26.6	4	<0.001
	LL	88	28.6	4	<0.001
	LW	88	41.7	4	<0.001

	SL	88	22.8	4	<0.001

References: n, sample size; d.f., degree of freedom; *H*, plant height with flowering culms; *Hw*, plant height without flowering culms; *LL*, 2nd leaf blade length; *LW*, 2nd leaf blade width, *ShL*, 2nd leaf sheath length; *NL*, 2nd internode length; *IL*, inflorescence length; *BRL*, basal raceme length; *EBR*, number of spikelets in the basal raceme; *NR*, raceme number; *VL*, flowering culms length with inflorescences; *SL*, spikelet length; *SW*, spikelet width; V*P*, extension of the vegetative period; *FP*, extension of the flowering period.

Table S3. Games-Howell post hoc test. Only traits that showed differentiation among populations are presented.

P. intermedium																
group1	group2	**BRL**	**EBR**	**FP**	**H**	**Hw**	**IL**	**LL**	**LW**	**NL**	**NR**	**SL**	**ShL**	**SW**	**VL**	**VP**
PIN2	PIN4	ns	**	****	****	****	****	****	****	****	****	***	****	-	-	-
PIN2	PIN7	**	****	****	****	****	**	****	ns	****	***	ns	*	-	-	-
PIN2	PIN8	ns	ns	ns	ns	***	ns	ns	ns	ns	***	*	ns	-	-	-
PIN2	PIN9	*	ns	****	***	ns	****	ns	ns	ns	****	***	****	-	-	-
PIN4	PIN7	****	ns	****	****	****	****	****	****	****	****	****	****	-	-	-
PIN4	PIN8	ns	**	****	****	****	****	***	****	****	****	****	****	-	-	-
PIN4	PIN9	ns	**	*	ns	****	ns	ns	****	****	ns	****	**	-	-	-
PIN7	PIN8	****	***	***	****	*	***	****	ns	****	****	***	ns	-	-	-
PIN7	PIN9	****	****	****	****	****	****	****	ns	****	****	****	***	-	-	-
PIN8	PIN9	ns	ns	****	****	***	****	ns	ns	*	****	ns	*	-	-	-
P. durifolium																
group1	group2	**BRL**	**EBR**	**FP**	**H**	**Hw**	**IL**	**LL**	**LW**	**NL**	**NR**	**SL**	**ShL**	**SW**	**VL**	**VP**
PD3	PD4	-	-	ns	-	-	-	ns	-	-	-	-	-	-	-	-
PD3	PD5	-	-	ns	-	-	-	ns	-	-	-	-	-	-	-	-
PD3	PD1	-	-	ns	-	-	-	ns	-	-	-	-	-	-	-	-
PD3	PD2	-	-	ns	-	-	-	ns	-	-	-	-	-	-	-	-
PD4	PD5	-	-	ns	-	-	-	**	-	-	-	-	-	-	-	-
PD4	PD1	-	-	*	-	-	-	**	-	-	-	-	-	-	-	-
PD4	PD2	-	-	ns	-	-	-	**	-	-	-	-	-	-	-	-
PD5	PD1	-	-	***	-	-	-	ns	-	-	-	-	-	-	-	-
PD5	PD2	-	-	ns	-	-	-	ns	-	-	-	-	-	-	-	-
PD1	PD2	-	-	*	-	-	-	ns	-	-	-	-	-	-	-	-
P. ionanthum																
group1	group2	**BRL**	**EBR**	**FP**	**H**	**Hw**	**IL**	**LL**	**LW**	**NL**	**NR**	**SL**	**ShL**	**SW**	**VL**	**VP**
PI3	PI4	-	-	*	ns	-	-	ns	-	ns	-	ns	****	ns	-	-

PI3	PI5	-	-	**	ns	-	-	ns	-	ns	-	ns	***	ns	-	-
PI3	PI1	-	-	*	ns	-	-	ns	-	ns	-	ns	ns	ns	-	-
PI3	PI2	-	-	ns	ns	-	-	ns	-	ns	-	**	**	***	-	-
PI4	PI5	-	-	****	ns	-	-	ns	-	ns	-	ns	ns	ns	-	-
PI4	PI1	-	-	ns	***	-	-	**	-	ns	-	ns	****	ns	-	-
PI4	PI2	-	-	*	*	-	-	ns	-	ns	-	ns	****	***	-	-
PI5	PI1	-	-	****	****	-	-	***	-	**	-	ns	****	ns	-	-
PI5	PI2	-	-	**	**	-	-	ns	-	*	-	ns	****	***	-	-
PI1	PI2	-	-	*	ns	-	-	**	-	ns	-	****	ns	***	-	-
P. regnellii																
group1	group2	**BRL**	**EBR**	**FP**	**H**	**Hw**	**IL**	**LL**	**LW**	**NL**	**NR**	**SL**	**ShL**	**SW**	**VL**	**VP**
PR2	PR1	-	**	****	ns	ns	-	-	-	-	-	-	*	-	ns	**
PR2	PR3	-	ns	*	ns	ns	-	-	-	-	-	-	ns	-	ns	ns
PR2	PR4	-	ns	ns	ns	*	-	-	-	-	-	-	ns	-	ns	ns
PR2	PR5	-	*	ns	**	*	-	-	-	-	-	-	ns	-	ns	ns
PR1	PR3	-	ns	****	ns	ns	-	-	-	-	-	-	*	-	ns	****
PR1	PR4	-	ns	****	ns	*	-	-	-	-	-	-	***	-	ns	****
PR1	PR5	-	ns	*	**	ns	-	-	-	-	-	-	ns	-	ns	**
PR3	PR4	-	ns	ns	ns	ns	-	-	-	-	-	-	ns	-	ns	ns
PR3	PR5	-	ns	ns	**	ns	-	-	-	-	-	-	ns	-	**	ns
PR4	PR5	-	ns	ns	**	ns	-	-	-	-	-	-	ns	-	**	ns
P. urvillei																
group1	group2	**BRL**	**EBR**	**FP**	**H**	**Hw**	**IL**	**LL**	**LW**	**NL**	**NR**	**SL**	**ShL**	**SW**	**VL**	**VP**
PU1	PU3	-	-	*	***	***	-	***	****	-	-	ns	-	-	-	-
PU1	PU4	-	-	*	*	****	-	***	****	-	-	ns	-	-	-	-
PU1	PU5	-	-	**	ns	ns	-	ns	**	-	-	ns	-	-	-	-
PU1	PU2	-	-	ns	ns	***	-	***	***	-	-	***	-	-	-	-
PU3	PU4	-	-	ns	ns	ns	-	ns	ns	-	-	ns	-	-	-	-

PU3	PU5	-	-	ns	ns	ns	-	ns	**	-	-	ns	-	-	-	-
PU3	PU2	-	-	ns	****	ns	-	ns	*	-	-	***	-	-	-	-
PU4	PU5	-	-	ns	ns	ns	-	ns	*	-	-	ns	-	-	-	-
PU4	PU2	-	-	ns	**	ns	-	ns	ns	-	-	*	-	-	-	-
PU5	PU2	-	-	ns	ns	ns	-	ns	ns	-	-	*	-	-	-	-

References: H, plant height with flowering culms; *Hw*, plant height without flowering culms; *LL*, 2nd leaf blade length; *LW*, 2nd leaf blade width, *ShL*, 2nd leaf sheath length; *NL*, 2nd internode length; *IL*, inflorescence length; *BRL*, basal raceme length; *EBR*, number of spikelets in the basal raceme; *NR*, raceme number; *VL*, flowering culms length with inflorescences; *SL*, spikelet length; *SW*, spikelet width; V*P*, extension of the vegetative period; *FP*, extension of the flowering period; -, traits that showed no significant differences among populations; ns, not significant; *$p<0.05$. **$p<0.01$. ***$p<0.001$. ****$p<0.0001$

Table S4. Annealing temperature of ISSR primers.

Primer	Annealing temperature	Primer	Annealing temperature
$(AC)_8T$	55ºC	$CAG(CA)_7$	55ºC
$(AC)_8G$	49ºC	$(CT)_8G$	50ºC
$(AG)_8T$	46ºC	$(CTC)_6AC$	60ºC
$(AG)_8C$	49ºC	$(GA)_8C$	49ºC
$(AG)_8GC$	50ºC	$(GA)_8T$	48ºC
$(AGAC)_4GC$	55ºC	$(GA)_8TC$	46ºC
$(ATG)_5GA$	46ºC	$(GAG)_7AC$	52ºC
$(CA)_8G$	49ºC	$(TC)_8A$	50ºC

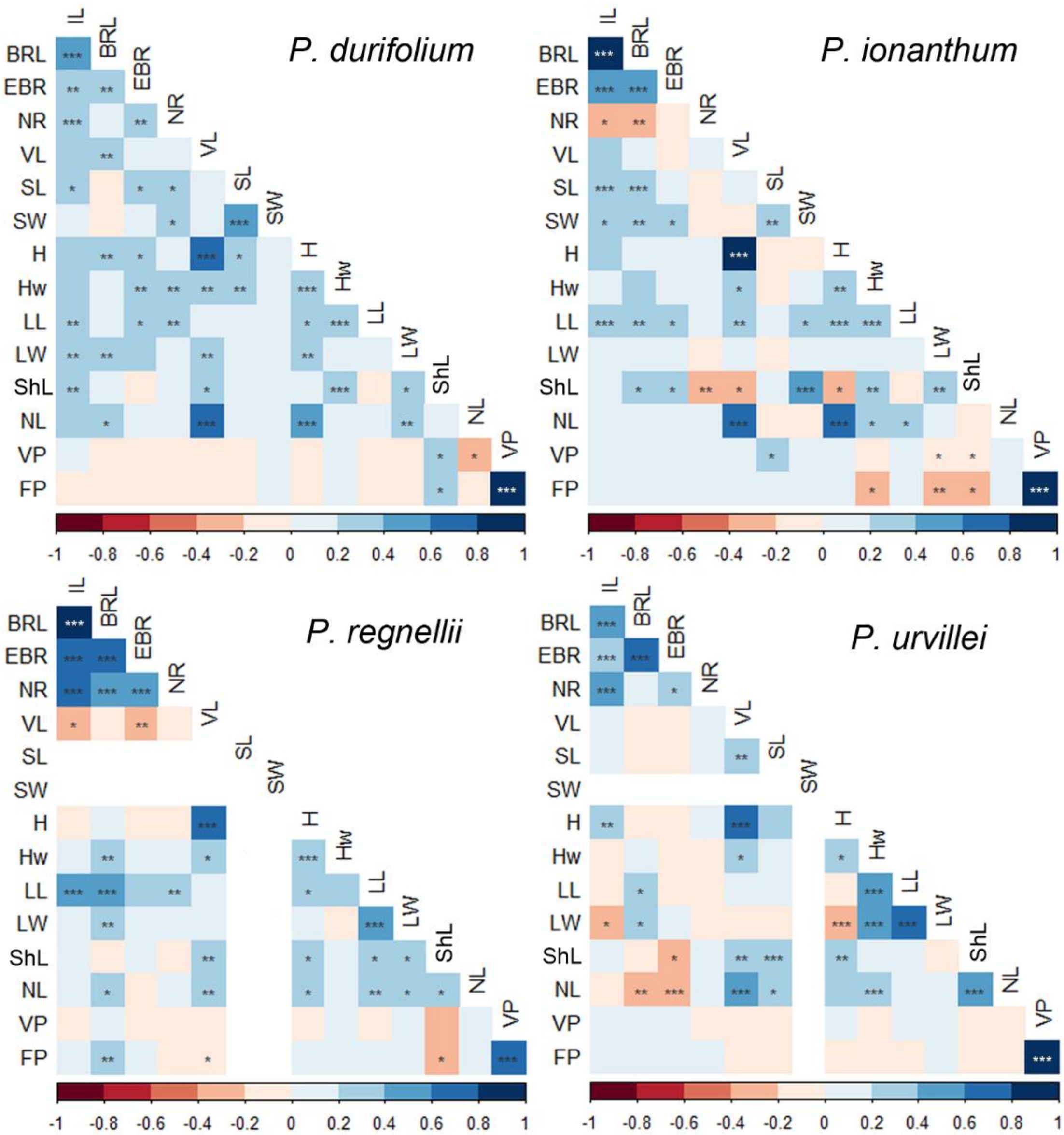

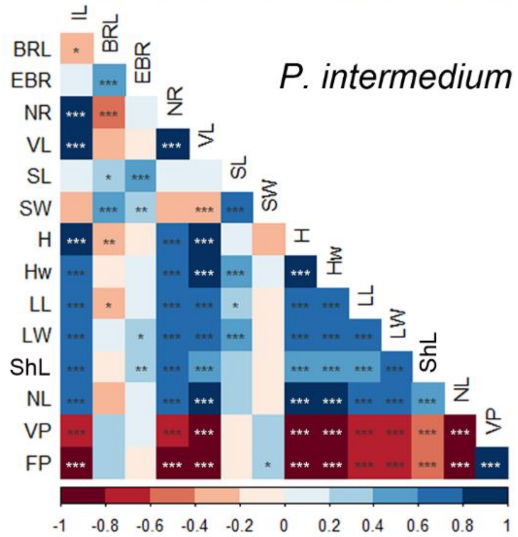

Figure S1. Correlation among phenotypic variables in each species. Color scheme indicates the correlation and asterisks the significance of the correlation. **p*<0.05, ***p*<0.01, ****p*<0.001. *H*, plant height with flowering culms; *Hw*, plant height without flowering culms; *LL*, 2nd leaf blade length; *LW*, 2nd leaf blade width, *ShL*, 2nd leaf sheath length; *NL*, 2nd internode length; *IL*, inflorescence length; *BRL*, basal raceme length; *EBR*, number of spikelets in the basal raceme; *NR*, raceme number; *VL*, flowering culms length with inflorescences; *SL*, spikelet length; *SW*, spikelet width; V*P*, extension of the vegetative period; *FP*, extension of the flowering period.

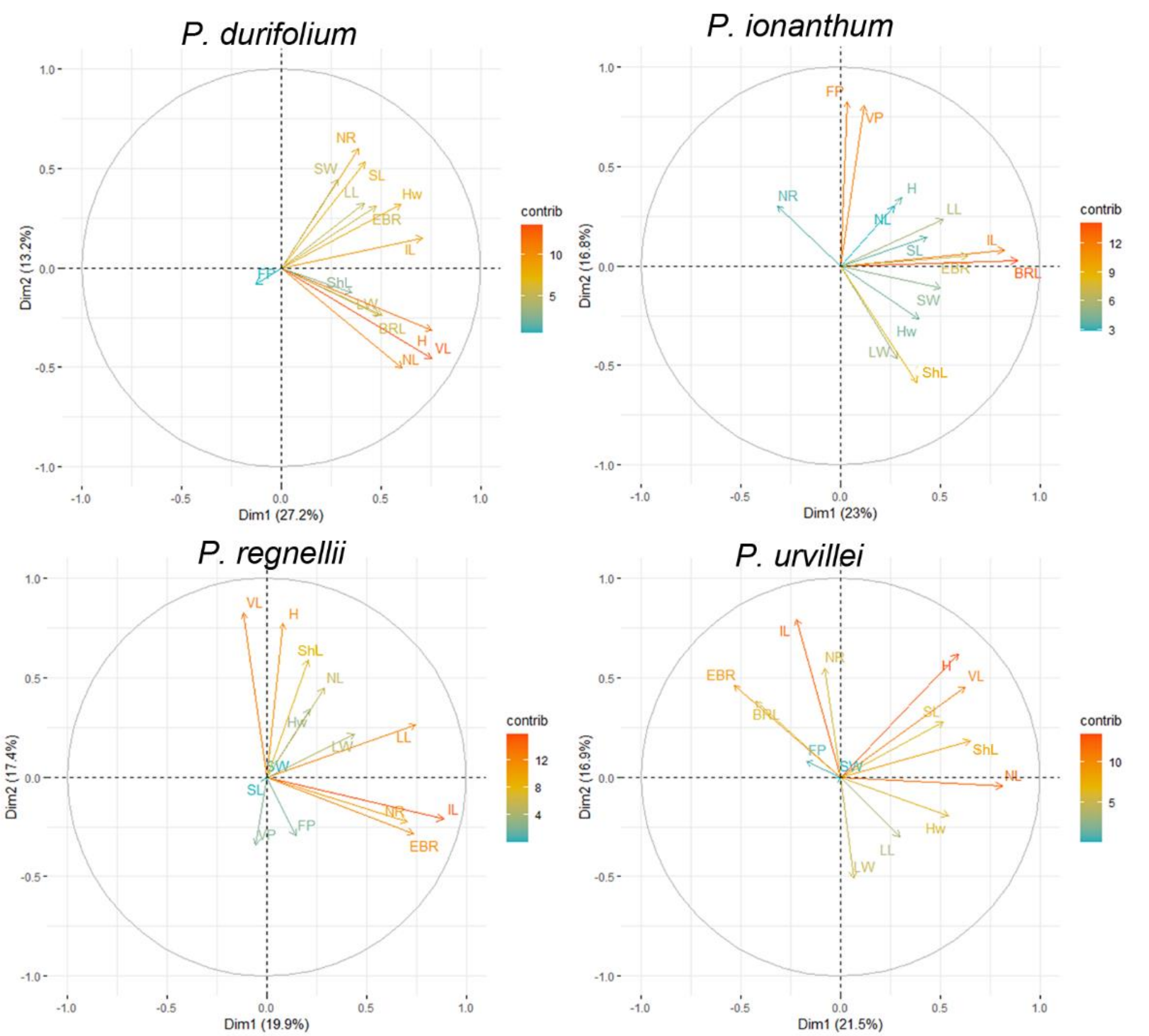

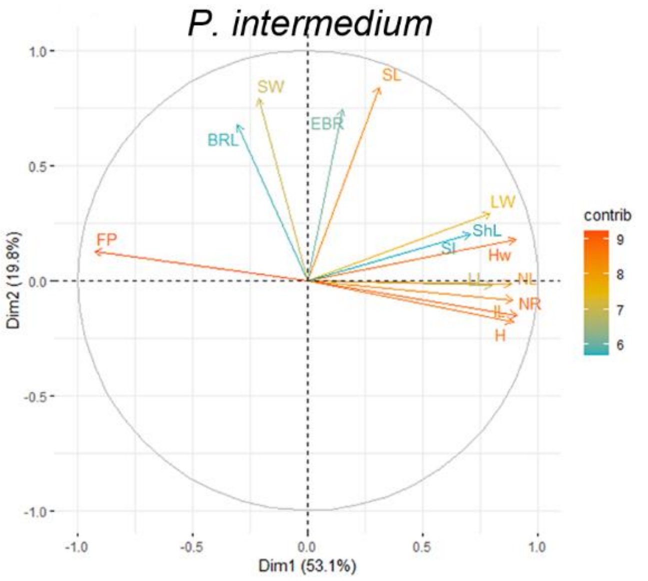

Figure S2. Contribution of each phenotypic variable to the PCA. Color scheme in legend represents the contribution of each trait to the principal components. *H*, plant height with flowering culms; *Hw*, plant height without flowering culms; *LL*, 2nd leaf blade length; *LW*, 2nd leaf blade width, *ShL*, 2nd leaf sheath length; *NL*, 2nd internode length; *IL*, inflorescence length; *BRL*, basal raceme length; *EBR*, number of spikelets in the basal raceme; *NR*, raceme number; *VL*, flowering culms length with inflorescences; *SL*, spikelet length; *SW*, spikelet width; V*P*, extension of the vegetative period; *FP*, extension of the flowering period.

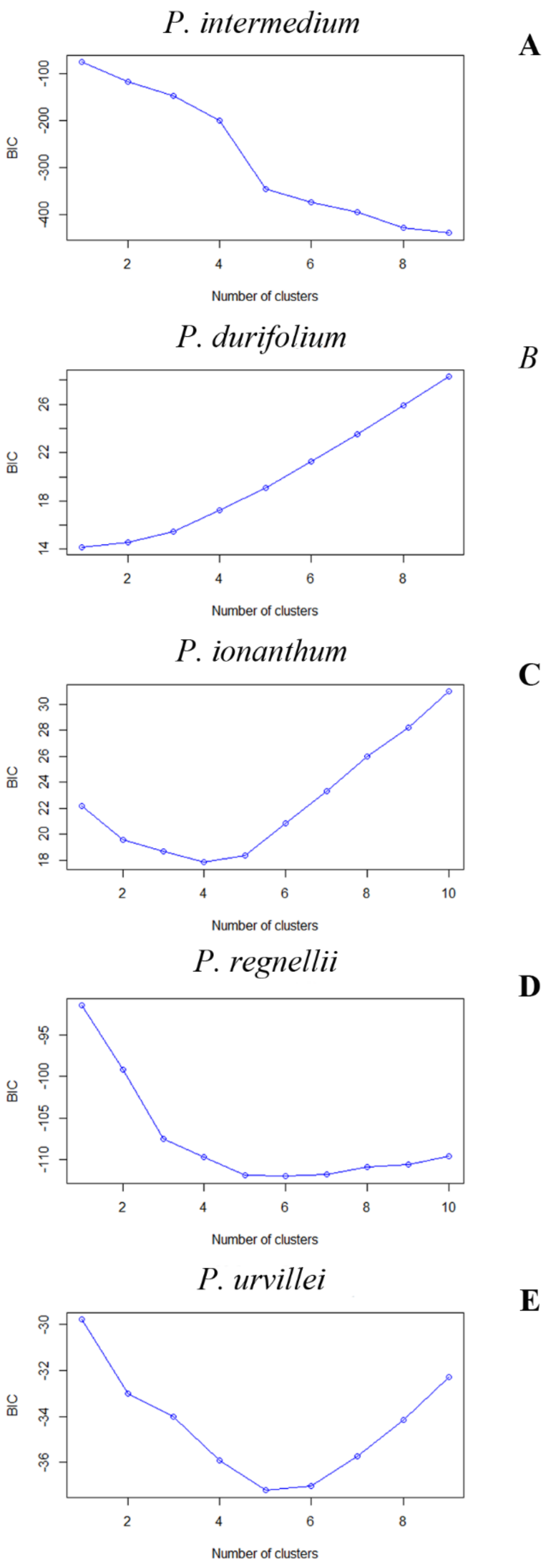

Figure S3. The effective number of clusters obtained using a k-means approach with the package *adegenet* in R.

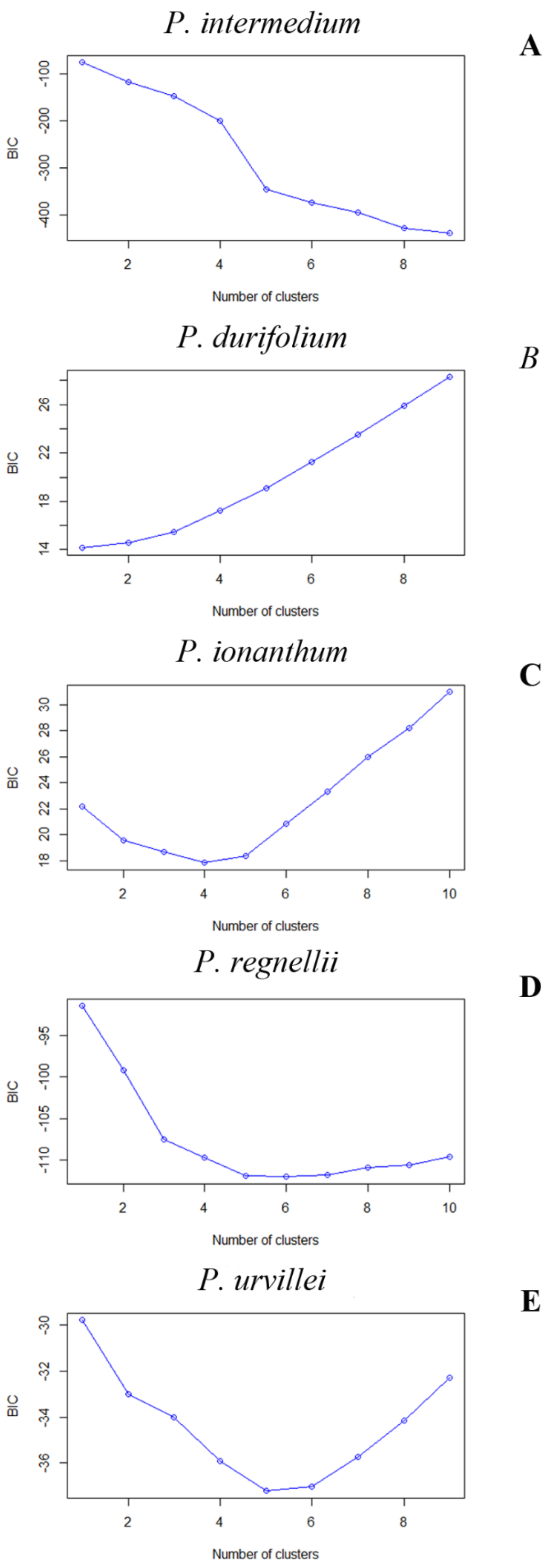

Figure S4. The effective number of clusters obtained for our dataset using a STRUCTURE-like approach with the package *LEA* in R**.**

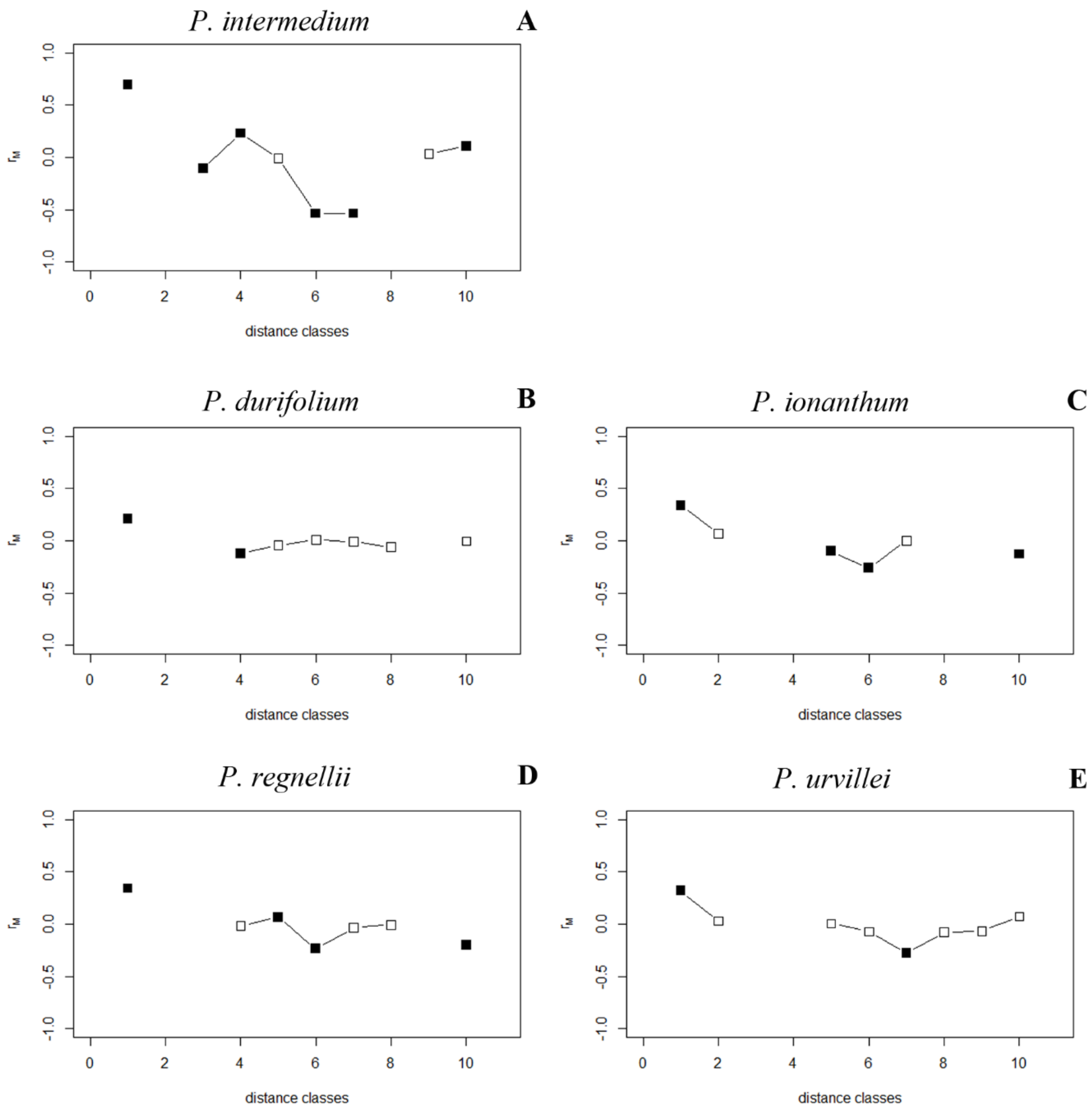

Figure S5. Identification of clusters isolated by distance using the *mpmcorrelogram* package in R. Those significantly isolated (p<0.05) are shown in black.
